# The Enhancement of the N1 Wave Elicited by Sensory Stimuli Presented at Very Short Inter-Stimulus Intervals Is a General Feature across Sensory Systems

**DOI:** 10.1371/journal.pone.0003929

**Published:** 2008-12-12

**Authors:** An Li Wang, André Mouraux, Meng Liang, Gian Domenico Iannetti

**Affiliations:** 1 Department of Physiology, Anatomy and Genetics, University of Oxford, Oxford, United Kingdom; 2 Centre for Functional Magnetic Resonance Imaging of the Brain, University of Oxford, Oxford, United Kingdom; Victoria University of Wellington, New Zealand

## Abstract

**Background:**

A paradoxical enhancement of the magnitude of the N1 wave of the auditory event-related potential (ERP) has been described when auditory stimuli are presented at very short (<400 ms) inter-stimulus intervals (ISI). Here, we examined whether this enhancement is specific for the auditory system, or whether it also affects ERPs elicited by stimuli belonging to other sensory modalities.

**Methodology and Principal Findings:**

We recorded ERPs elicited by auditory and somatosensory stimuli in 13 healthy subjects. For each sensory modality, 4800 stimuli were presented. Auditory stimuli consisted in brief tones presented binaurally, and somatosensory stimuli consisted in constant-current electrical pulses applied to the right median nerve. Stimuli were delivered continuously, and the ISI was varied randomly between 100 and 1000 ms. We found that the ISI had a similar effect on both auditory and somatosensory ERPs. In both sensory modalities, ISI had an opposite effect on the magnitude of the N1 and P2 waves: the magnitude of the auditory and the somatosensory N1 was significantly increased at ISI≤200 ms, while the magnitude of the auditory and the somatosensory P2 was significantly decreased at ISI≤200 ms.

**Conclusion and Significance:**

The observation that both the auditory and the somatosensory N1 are enhanced at short ISIs indicates that this phenomenon reflects a physiological property that is common across sensory systems, rather than, as previously suggested, unique for the auditory system. Two of the hypotheses most frequently put forward to explain this observation, namely (i) the decreased contribution of inhibitory postsynaptic potentials to the recorded scalp ERPs and (ii) the decreased contribution of ‘latent inhibition’, are discussed. Because neither of these two hypotheses can satisfactorily account for the concomitant reduction of the auditory and the somatosensory P2, we propose a third, novel hypothesis, consisting in the modulation of a single neural component contributing to both the N1 and the P2 waves.

## Introduction

Brief sensory stimuli can elicit transient responses (event-related potentials, ERPs) in the ongoing electroencephalogram (EEG) [1]. The largest part of these responses is constituted by a biphasic negative-positive wave (N1-P2), maximal at the vertex [2], [3], [4], [5], [6]. It is commonly observed that the magnitude of the N1-P2 response elicited by stimuli repeated at constant inter-stimulus interval (ISI) is strongly dependent on the repetition rate (reviewed in [7]): the shorter the ISI, the smaller the magnitude of the N1-P2 response [4], [8], [9]. This phenomenon is usually explained in terms of refractoriness of the neural generators underlying the N1-P2 response [5], [10], [11], [12], [13], [14], [15]. However, in striking contradiction with this explanation, it has been reported that when auditory stimuli are presented using a variable and very short ISI, the direction of this modulation is changed: at ISIs<400 ms, stimulus repetition actually *increases* the magnitude of the auditory N1. Using magnetoencephalography (MEG), Loveless et al. [16] were the first to observe that at randomly varying ISIs ranging from 150 to 230 ms, the magnitude of the auditory N100m (the magnetic counterpart of the auditory N1 wave recorded using EEG) was significantly enhanced. Further evidence from both MEG [16], [17], [18] and EEG [19], [20] experiments have confirmed this observation. For example, Budd et al. [19] recorded auditory ERPs elicited by a train of auditory stimuli presented using an ISI randomly varied between 100 and 1000 ms, and found that the amplitude of the auditory N1 was increased at ISIs ranging from 100 to 300 ms. This phenomenon has been labelled ‘N1 enhancement’ or ‘N1 facilitation’, and has been interpreted as reflecting an increased activity of the neural generators underlying the auditory N1, due either to a change in the respective contribution of excitatory and inhibitory postsynaptic potentials [16], or to a mechanism of ‘latent inhibition’ [16], [18], [21], [22]. So far, the effect of stimulus repetition at very short ISI on the N1-P2 response has been investigated only in the auditory modality. Therefore, whether it leads to a similar enhancement of the N1 wave elicited by stimuli belonging to other sensory modalities is unknown.

**Figure 1:**
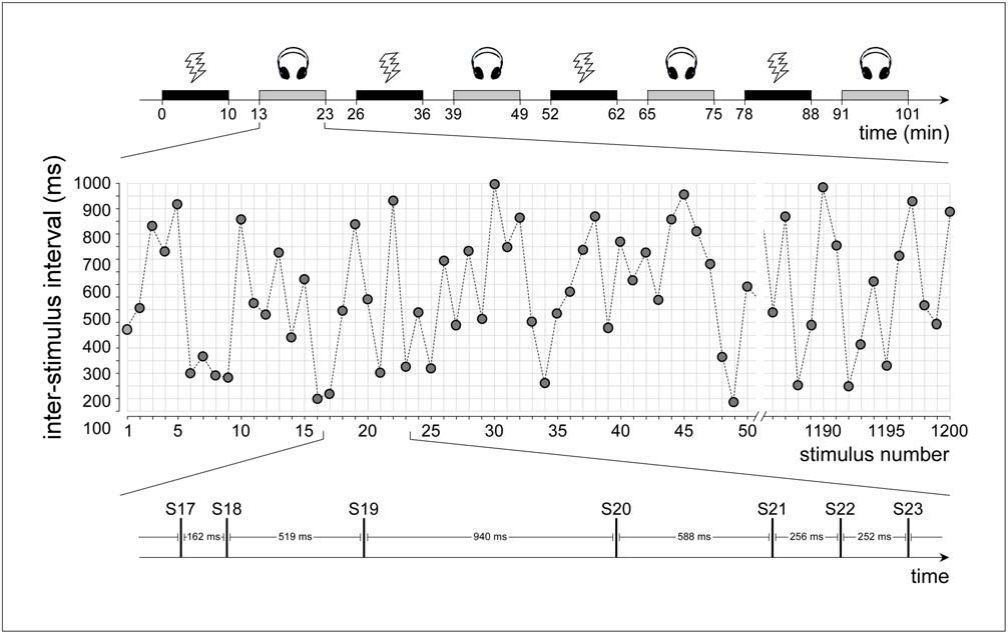
Experimental paradigm. EEG data was collected in a single session. Within this session, four blocks of auditory (grey) and four blocks of somatosensory (black) stimulation were presented in alternation (top panel). Each block lasted approximately 11 minutes, and consecutive blocks were separated by a 3-minute break. Auditory stimuli consisted in brief 800 Hz tones delivered binaurally through headphones, and somatosensory stimuli consisted in electrical pulses delivered to the right median nerve through surface electrodes. In each block (middle panel), 1200 identical stimuli were delivered, and the inter-stimulus interval was randomly varied from trial to trial between 100 and 1000 ms (bottom panel).

Hence, the following question is still unaddressed: does the enhancement of the auditory N1 at very short ISI reflect, as previously suggested [19], [22], an auditory-specific mechanism, or does it reflect a physiological mechanism common across sensory modalities? In order to address this question, we recorded ERPs elicited by auditory and somatosensory stimuli delivered continuously using an ISI randomly varied between 100 and 1000 ms (Figure 1).

## Results

### Auditory ERPs

**Figure 2:**
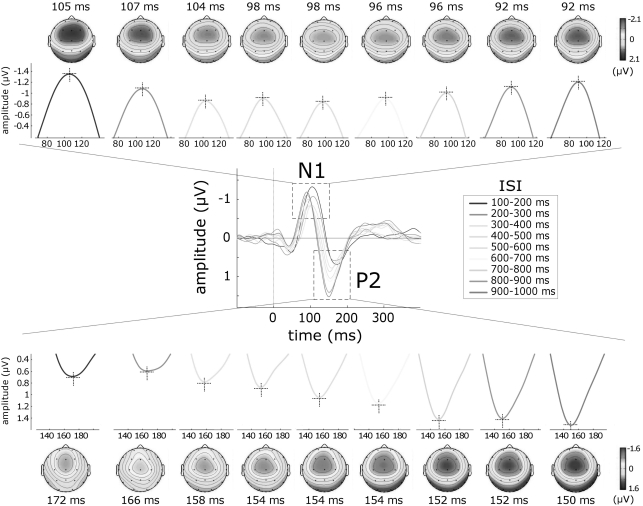
Effect of inter-stimulus interval (ISI) on the auditory N1 and P2 waves. Trials were classified according to ISI, yielding nine categories ranging from 100 to 1000 ms in steps of 100 ms. At short ISIs, the brain activity elicited by two consecutive stimuli was likely to overlap and therefore distort the obtained ERP waveform. This distortion was corrected using the Adjacent Response procedure [35] (see Methods). The *middle panel* displays the auditory ERP obtained at each ISI category (group-level average; Cz vs. average reference). Each ISI category is colour coded. *x* axis, time (ms); *y* axis, amplitude (µV). *Upper* and *lower panels* display the N1 and P2 waves and their scalp distributions, separately for each ISI. Note the opposite effect of ISI on the amplitude of the auditory N1 and P2 waves: at very short ISIs, the N1 displays significantly larger amplitudes, while the P2 displays significantly smaller amplitudes.

The group-level average waveforms of auditory ERPs and the scalp distributions of the auditory N1 and P2 waves are displayed in Figure 2 (n = 13).

**Figure 3 pone-0003929-g003:**
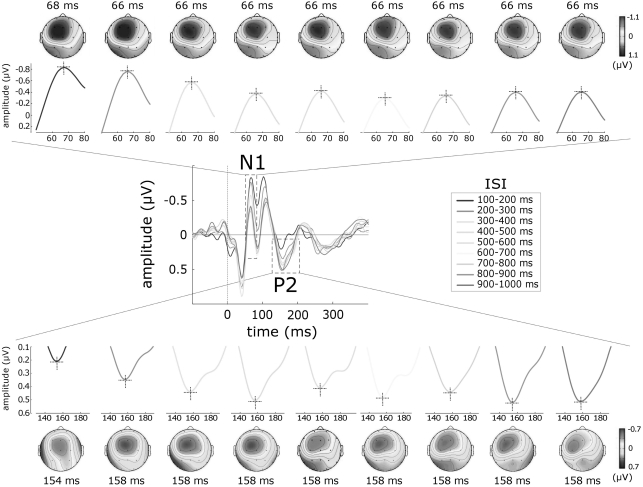
Effect of inter-stimulus interval (ISI) on the average amplitude of the auditory and somatosensory N1 and P2 waves. *y* axis: amplitude (µV); *x* axis: ISI category. Both in the auditory and in the somatosensory modality the ISI had an opposite effect on the auditory and somatosensory N1 and P2: at shorter ISIs, the N1 displayed significantly larger amplitudes while the P2 displayed significantly smaller amplitudes. Error bars represent the variance across subjects (standard error of the mean). Asterisks highlight ISI categories in which the average peak amplitude was significantly different from the peak amplitude at the category ‘100–200 ms’ (* p<0.05; ** p<0.01).

The amplitude of both the N1 and the P2 wave was significantly affected by the ISI, but in opposite directions: while the amplitude of the N1 wave was significantly larger at short ISIs (p<.001), the amplitude of the P2 wave was significantly smaller at short ISIs (p<.0001) (Figure 3, left panel). Post-hoc comparisons revealed that N1 amplitude at ISI category 100–200 ms was significantly larger than N1 amplitude at all ISI categories between 300 and 800 ms (p<.01). On the contrary, P2 amplitude at ISI category 100–200 ms was significantly smaller than P2 amplitude at all ISI categories between 600 and 1000 ms (p<.001). The latency of the auditory N1 and the latency of the auditory P2 were also significantly affected by the ISI (p<.0001). Post-hoc comparisons revealed that N1 latency at ISI category 100–200 ms was significantly longer than N1 latency at all ISI categories between 400 and 1000 ms (p values ranging from <.05 to <.001). Similarly, post-hoc comparisons revealed that P2 latency at ISI category 100–200 ms was significantly longer than P2 latency at all ISI categories between 400 and 1000 ms (p values ranging from <.01 to <.001).

### Somatosensory ERPs

**Figure 4:**
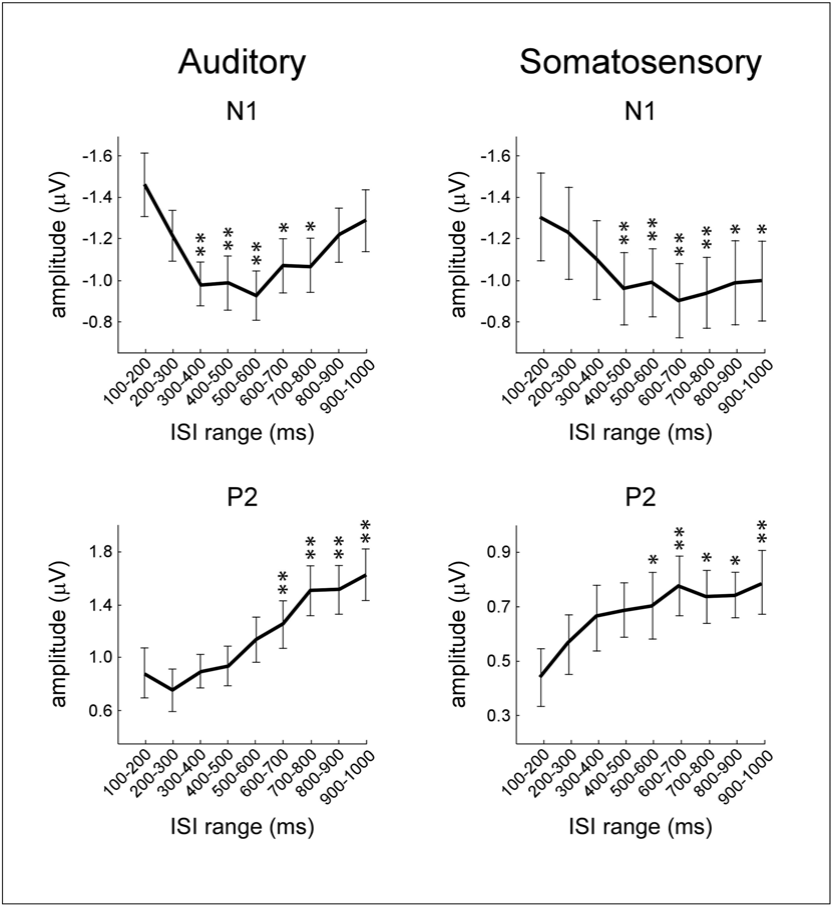
Effect of inter-stimulus interval (ISI) on the somatosensory N1 and P2 waves. Trials were classified according to ISI, yielding nine categories ranging from 100 to 1000 ms in steps of 100 ms. Response overlap was corrected using the Adjacent Response procedure [35] (see Methods). The *middle panel* displays the somatosensory ERP obtained at each ISI category (group-level average; P3 vs. average reference). Each ISI category is colour coded. *x* axis, time (ms); *y* axis, amplitude (µV). *Upper* and *lower panels* display the N1 and P2 waves and their scalp distributions, separately for each ISI. Note the opposite effect of ISI on the amplitude of the somatosensory N1 and P2 waves: at very short ISIs, the N1 displays significantly larger amplitudes, while the P2 displays significantly smaller amplitudes.

Two subjects were excluded from the analysis, due to the lack of any identifiable somatosensory ERP. The group-level average waveforms of somatosensory ERPs and the scalp distributions of the N1 and P2 waves are displayed in Figure 4 (n = 11).

Similarly to what was observed in the auditory modality, the amplitude of the somatosensory N1 and the amplitude of the somatosensory P2 were both significantly affected by the ISI, but in opposite directions: the amplitude of the N1 wave was significantly larger at short ISIs (p<.0001), while the amplitude of the P2 wave was significantly smaller at short ISIs (p<.001) (Figure 3, right panel). Post-hoc comparisons revealed that N1 amplitude at ISI category 100–200 ms was significantly larger than N1 amplitude at all ISI categories between 400 and 1000 ms (p values ranging from <.05 to <.001). On the contrary, P2 amplitude at ISI category 100–200 ms was significantly smaller than P2 amplitude at all ISI categories ranging from 500 to 1000 ms (p values ranging from <.05 to <.01). The latency of the somatosensory N1 and the latency of the somatosensory P2 were not affected by ISI (p>.05).

## Discussion

This study shows that stimulus repetition at very short ISIs (100–1000 ms) similarly affects the amplitude of both auditory and somatosensory ERPs: at ISIs≤200 ms the N1 wave displays significantly larger amplitudes, while the P2 wave displays significantly smaller amplitudes (Figures 2–4).

**Figure 5:**
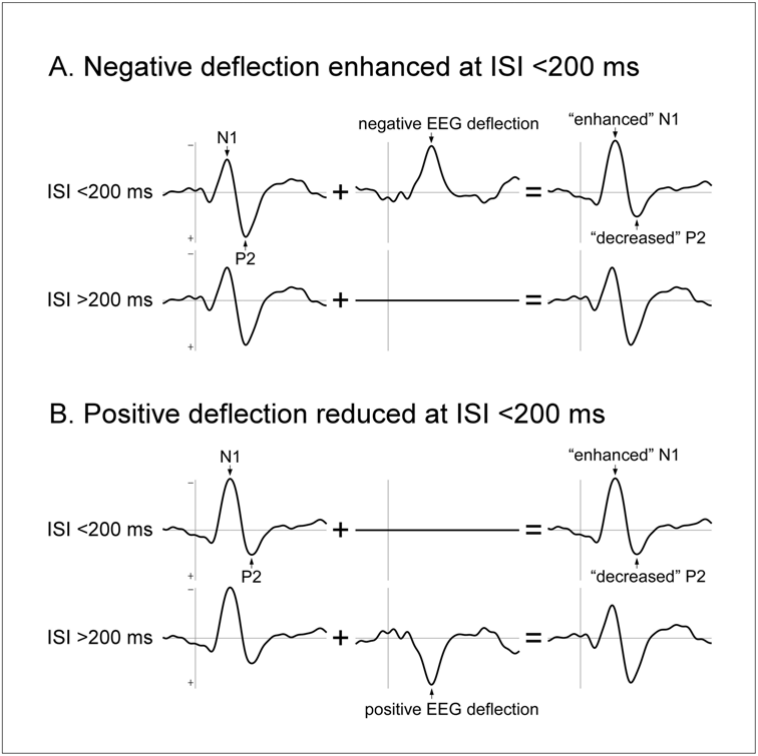
A novel hypothesis to explain the effect of inter-stimulus interval (ISI) on the amplitude of the N1 and P2 waves. The effect of ISI on the amplitude of the auditory and somatosensory N1 and P2 could be explained by the modulation of a single neural component whose time course overlaps the peak latency of the N1 and P2 waves (middle column). This component could appear in the EEG either (A) as a negative deflection that is *enhanced* at very short ISIs, or (B) as a positive deflection that is *reduced* at very short ISIs. In both cases, at very short ISIs the magnitude of the N1 would be increased and the magnitude of the P2 would be decreased (right column). Note that in this model, the neural components underlying the N1 and P2 waves *per se* are not modulated by ISI (left column).

The finding that the ISI-dependent enhancement of the auditory N1 also affects the somatosensory N1 indicates that this phenomenon reflects a physiological mechanism common across sensory modalities, rather than, as suggested previously [19], [22], a mechanism specific for the auditory system. In addition, the observation of a concomitant reduction of both the auditory and the somatosensory P2 indicates that the N1 enhancement, which has been previously explained in terms of facilitation of a subset of its underlying generators [16], [19], could alternatively be explained by a modulation of the magnitude of a single neural component whose contribution to the scalp ERP overlaps both the N1 and the P2 wave [22]. This component could appear in the scalp EEG either as a positive deflection that is *reduced* at very short ISIs, or as a negative deflection that is *enhanced* at very short ISIs, thus increasing the magnitude of the N1 wave and decreasing the magnitude of the P2 wave at very short ISI (figure 5).

### ISI-dependent enhancement of the auditory and somatosensory N1 wave

The observed enhancement of the auditory N1 at ISIs shorter than 200 ms is consistent with the findings of previous studies, conducted using both EEG [19], [20] and MEG [16], [17], [18]. Budd et al. [19] and Todd et al. [20] recorded ERPs elicited by a train of auditory stimuli presented at randomly varying ISIs ranging from 50 to 1000 ms, and found that the amplitude of the auditory N1 was increased at ISIs shorter than 300 ms and 150 ms, respectively. The increase was located over fronto-central scalp electrodes. These observations match well our current results, both in terms of the ISI at which the enhancement is observed, and of its scalp distribution (Figure 2). Most importantly, our results show for the first time that also the somatosensory N1 is enhanced at very short ISIs. Because the N1 enhancement has been previously observed only in the auditory modality, it had been mainly interpreted as reflecting a mechanism specific for the auditory system, and explained in terms of, for example, a facilitation of discrete areas in the primary auditory cortex [19]. Our observation that a similar enhancement also affects the somatosensory N1 (Figures 3, 4) indicates that this phenomenon reflects a mechanism that is common across sensory systems, either affecting similarly the responsiveness of auditory-specific and somatosensory-specific cortical areas respectively contributing to the auditory and somatosensory N1, or affecting multimodal cortical areas contributing equally to the generation of both waves. In support of the latter hypothesis, there is evidence that a significant part of the N1 peak elicited by stimuli of different sensory modalities (e.g. auditory, somatosensory and visual) reflects neural activities that are elicited by environmental stimuli regardless of their sensory modality [2], [23], [24].

In addition, our results show that stimulus repetition at ISI≤200 ms induces not only an enhancement of the N1 wave, but also a concomitant reduction of the P2 wave (Figure 5). This finding must be taken into account when discussing the possible neurophysiological mechanisms underlying the effect of stimulus repetition at very short ISIs.

To explain the enhancement of the auditory N1 induced by stimulus repetition at very short ISIs, two main hypotheses have been put forward: (i) a decreased contribution of inhibitory postsynaptic potentials to the recorded response [16], and (ii) a mechanism of ‘latent inhibition’ [16], [18], [21], [22].

#### (i) Decreased contribution of inhibitory postsynaptic potentials

It is well established that ERPs mostly reflect summed postsynaptic potentials originating from a large population of synchronously activated neurons [25]. Afferent sensory stimulation causes an initial excitatory postsynaptic potential (EPSP) in cortical pyramidal cells, followed by a longer-lasting inhibitory postsynaptic potential (IPSP). Consequently, the evoked potentials recorded from the scalp result from the opposite interaction between EPSPs and IPSPs, and the N1 enhancement observed at very short ISIs could thus result from a selective reduction of the contribution of IPSPs [16]. In agreement with this first hypothesis, Deisz and Prince [26], performing intracellular recordings of *in vitro* preparations of guinea-pig neocortical slices, found that when the rate of stimulation is increased, the magnitude of elicited IPSPs diminishes more rapidly than the magnitude of elicited EPSPs. Furthermore, Nacimiento et al. [27] showed that IPSPs completely disappear when the eliciting stimuli are presented at ISIs shorter than 200 ms.

#### (ii) Latent inhibition

The second hypothesis is that the observed enhancement of the N1 wave results from a mechanism of ‘latent inhibition’ [18], [22]. According to this model, a sensory afferent volley evokes a large excitatory response in the neural population generating the N1 wave. As this primary response spreads through association fibres, it would elicit a less precisely time-locked secondary excitatory response in neighbouring inhibitory interneurons which, in turn, would exert a long-lasting inhibition on the neural population generating the N1 wave [18]. In other words, the initial excitation of N1 generators would spread to neurons that, in turn, feedback on the N1 generators and inhibit subsequent N1 responses. Because this inhibitory feedback mechanism would require time to build up, a second stimulus arriving while inhibition is still latent (e.g. ISI<400 ms) would produce a larger response than a second stimulus arriving after inhibition has taken place (e.g. ISI>400 ms).

However, both the ‘EPSP/IPSP unbalance’ and the ‘latent inhibition’ hypotheses would predict that stimulus repetition at short ISIs leads to a similar enhancement of the later auditory and somatosensory P2 waves, while our results show the opposite, i.e. that the magnitude of the auditory and the somatosensory P2 is significantly reduced when stimuli are presented at very short ISIs (Figure 3).

Therefore, to account fully for the present results, we propose a third, novel hypothesis: that stimulus repetition at very short ISIs modulates the activity of a single neural component whose time course overlaps the peak latency of the N1 and P2 waves. This component would appear in the EEG either as a negative deflection that is enhanced at very short ISIs, or as a positive deflection that is reduced at very short ISIs, thus concomitantly increasing the magnitude of the N1 and decreasing the magnitude of the P2 (Figure 5). Which kind of stimulus-evoked neural activity could fit this description? Numerous studies have shown that deviant auditory stimuli presented within a constant stream of repeated auditory stimuli elicit a “mismatch negativity” (MMN), consisting of a long-lasting negative deflection, typically peaking at 150–250 ms after stimulus onset, and overlapping both the N1 and P2 waves [5], [28]. The neural generators underlying the MMN have been hypothesized to be independent from the neural generators underlying the N1 and P2, and are usually interpreted as reflecting brain processes triggered when an incoming stimulus *mismatches* the memory representation formed by the preceding stimulus. Therefore, considering that the formation of this memory trace requires a certain amount of time to be established, it could be that, at very short ISIs, stimuli elicit a MMN because the memory representation of the preceding stimulus has not had enough time to form itself (Figure 5, upper panel), or because the latency at which the stimulus occurred strongly deviated from the mean ISI [22]. Both hypotheses would agree with our observation that stimulus repetition similarly affected auditory and somatosensory ERPs. Indeed, several studies have shown that a response similar to the auditory MMN can be elicited by stimuli belonging to other sensory modalities [29], [30], [31]. Interestingly, Haenschel et al. recently showed that the formation of a memory representation is associated with a “repetition positivity”, consisting in a fronto-central positive deflection in the EEG occurring between 50 and 250 ms after stimulus onset [32]. Hence, an alternative explanation of our finding could be that at very short ISIs, the formation of this memory trace is disrupted, thus leading to a reduced contribution of this positive deflection (Figure 5, lower panel).

In conclusion, our results show that the enhancement of the N1 wave observed at very short ISIs reflects a physiological property that is common for the processing of auditory and somatosensory input, and thus that it is not unique for the auditory system.

Furthermore, by showing that the enhancement of the N1 wave is associated with a concurrent attenuation of the subsequent P2 wave, our results indicate that the N1 enhancement does not result necessarily from the enhancement of the cortical generators underlying this N1 wave. Instead, the previously described N1 enhancement could reflect the modulation of a single neural component whose time course overlaps the peak latency of the N1 and the P2 wave. This component, which would appear in the EEG either as a negative deflection that is enhanced at very short ISIs (possibly related to the “mismatch negativity”, [28]), or as a positive deflection that is reduced at very short ISIs (possibly related to the “repetition positivity”, [32]), would concomitantly increase the N1 magnitude and decrease the P2 magnitude.

Therefore, while we do not refute the possibility suggested by several authors that the enhancement of the N1 wave observed at very short ISIs reflects an enhancement of the neural activity underlying its generation, we believe that this alternative hypothesis should be considered when interpreting the modulation of ERPs elicited by stimulus repetition.

## Materials and Methods

### Subjects

Thirteen healthy volunteers (six females and seven males) aged 23–36 years participated in the study. The subjects were recruited from research staff and students of the University of Oxford (UK). All participants gave their written informed consent after all the experimental procedures were explained. The study was approved by the local ethics committee.

### Stimuli

Auditory stimuli were brief 800 Hz tones of 30 ms duration (5 ms rise and fall times; ∼80 dB SPL) delivered binaurally through headphones (Sennheiser, HD202, Germany). Somatosensory stimuli were constant current square-wave electrical pulses of 500 µs duration generated by a DS7A Constant Current Stimulator (Digitimer Ltd, UK). Electrical pulses were delivered through a bipolar electrode (1 cm inter-electrode distance) placed at the wrist, over the right median nerve. The intensity of the electrical stimulus was adjusted in each subject, just above the threshold to elicit a twitch of the thumb (4.8±2.4 mA).

### Experimental paradigm

A scheme of the experimental paradigm is shown in Figure 1. EEG data was collected in a single session. Within this session, four blocks of auditory and four blocks of somatosensory stimuli were presented in alternation (eight blocks in total). The order of the blocks was balanced across subjects. Each block lasted approximately 11 minutes, and consisted of a train of 1200 identical stimuli presented with an ISI that varied randomly between 100 and 1000 ms (rectangular distribution). Blocks were separated by a resting period of approximately 3 minutes.

### EEG recording

Participants were seated in a comfortable chair, and were instructed to read quietly whilst relaxing their muscles and minimising eye movements and blinks. The electroencephalogram (EEG) was recorded using 19 scalp Ag-AgCl electrodes, placed according to the international 10–20 system, using the nose as extracephalic reference. Signals were amplified and digitised using a sampling rate of 512 Hz and a conversion of 12 bit, giving a resolution of 0.195 µV digit^−1^ (System Plus; Micromed, Treviso, Italy). To monitor ocular movements and eye blinks, the electro-oculogram was recorded using two surface electrodes, one placed over the lower eyelid, the other placed lateral to the lateral corner of the orbit. In addition, the electrocardiogram was recorded using two surface electrodes placed at the left and right forearms, midway between the wrist and the elbow.

### EEG data analysis

Analysis of the EEG data was performed using Letswave (http://amouraux.webnode.com/letswave) [33], Matlab (The MathWorks, USA) and EEGLAB (http://sccn.ucsd.edu/eeglab). Continuous EEG recordings were segmented into 3-second-long epochs (from −1 to +2 s relative to stimulus onset), filtered (2–30 Hz band-pass filter), re-referenced using an average reference, and baseline-corrected (baseline interval −1 to 0 s relative to stimulus onset). Artifacts produced by eye blinks and eye movements were subtracted using a validated method based on Independent Component Analysis [34]. In all datasets, individual eye movements could be seen clearly in the independent components (IC) removed (4±1 ICs). In addition, epochs with amplitude values exceeding 100 µV were rejected from further analysis. Epochs were then classified in nine categories (ranging from 100 to 1000 ms in steps of 100 ms) according to the duration of the preceding ISI (i.e. the duration of the interval separating the onset of the stimulus and the onset of the preceding stimulus). Separate ERP average waveforms were computed for each stimulus modality and each of the nine categories, thus yielding 18 average waveforms for each participant. Because of the short ISI used in this experiment, the brain responses elicited by two consecutive stimuli were likely to overlap and therefore distort the computed ERP waveforms. This response overlap was corrected using a validated procedure named Adjacent Response (Adjar level 1; [35]). The procedure consists in the following four steps, all performed at single-subject level. (i) A relatively undistorted ERP waveform (the “full average”, [35]) is obtained by averaging all the 4800 trials independently of ISI. (ii) An ERP waveform distorted by the activity elicited by the preceding stimulus is obtained by averaging the trials belonging to each ISI category (∼530 stimuli per category). (iii) For each category, a waveform representing an estimation of the distortion due to the overlap with the responses elicited by the preceding stimulus (the “previous-response overlap”, [35]) is obtained by averaging the response evoked by each preceding trial (estimated using the “full average” waveform), shifted according to the actual ISI value of each trial. (iv) Finally, the “previous-response overlap” waveform is subtracted from the distorted ERP waveform, thus yielding an estimation of the ERP for each ISI category, corrected for the distortion due to the response to the preceding stimulus.

The peak latency and the baseline-to-peak amplitude of auditory and somatosensory N1 and P2 waves were measured for each subject and ISI category using the average waveforms obtained from the Adjar procedure. In the auditory ERPs, N1 and P2 waves were identified at the vertex (Cz). The auditory N1 was defined as the largest negative deflection occurring between 80 and 120 ms after stimulus onset [5]. The auditory P2 was defined as the largest positive deflection occurring between 140 and 200 ms after stimulus onset [36]. In the somatosensory ERPs, the N1 and P2 waves were identified at channel P3 [14], [37]. The somatosensory N1 was defined as the largest negative deflection following stimulus onset. The somatosensory P2 was defined as the largest positive deflection occurring between 130 and 200 ms after stimulus onset. All values are given as arithmetic mean±standard error of the mean.

### Statistical analysis

As all measured amplitude and latency values were distributed normally (D'Agostino-Pearson normality test), differences in latency and amplitude between the nine ISI categories were assessed using a repeated-measure one-way ANOVA. When the means were significantly different (p<.05), the nine ISI categories were compared using a post-hoc Tukey's test. All statistical analyses were conducted using Prism 5.0 (Graphpad, USA).
